# Predicting Psychological Resilience in Older Adults During the COVID-19 Pandemic: A Machine Learning Approach

**DOI:** 10.1093/geroni/igaf122.4176

**Published:** 2025-12-31

**Authors:** Josephine Abrials, Xuan Lu, Xiwen Guan, Xiaoling Xiang

**Affiliations:** University of Michigan, Ann Arbor, Michigan, United States; University of Arizona, Tucson, Arizona, United States; University of Michigan, Ann Arbor, Michigan, United States; University of Michigan-Ann Arbor, Ann Arbor, Michigan, United States

## Abstract

Research has demonstrated that resilience is the norm after disasters, yet not all individuals and communities adapt equally well. Studies on resilience have predominantly been cross-sectional and relied on traditional statistical methods. Adopting a longitudinal design, this study aimed to predict psychological resilience among older adults during the COVID-19 pandemic using machine learning methods. The study sample consisted of 3,364 individuals who completed the 2016 and 2020 Leave-Behind Questionnaire from the Health and Retirement Study. A comprehensive, theory-informed set of predictors at the individual, interpersonal, and community levels was measured in 2016, and resilience was measured using a 6-item scale in 2020 (Cronbach’s α = .81). Three machine learning algorithms (LASSO, Ridge, and Random Forest) were trained with five-fold cross-validation, with LASSO being the best fit to the data (RMSE = 0.873; R² = 0.195). SHAP values were used to interpret feature importance. Twenty-four important predictors emerged, including psychological traits (e.g., optimism, openness), pre-pandemic social participation, social support, and neighborhood cohesion, as well as indicators of technology adaptation (e.g., learning a new device) during the pandemic. Non-linear and interaction effects were also identified. Study findings highlight the complex and multifaceted nature of resilience, demonstrating the value of a theory-informed data science approach. Addressing digital inequities and fostering social support and participation are potential targets for resilience promotion. Future studies could build upon our findings and employ causal inference methods to better understand the causes and mechanisms that promote psychological resilience in older adults.

